# The Challenge of Prenatal Diagnostic Work-Up of Maternally Inherited X-Linked Opitz G/BBB: Case Report and Literature Review

**DOI:** 10.1155/2015/830108

**Published:** 2015-05-04

**Authors:** Marialuigia Spinelli, Carmine Sica, Bruno Dallapiccola, Antonio Novelli, Letizia Di Meglio, Pasquale Martinelli

**Affiliations:** ^1^Department of Neurosciences, Reproductive Sciences and Odontostomatoloy, University of Naples Federico II, 80100 Naples, Italy; ^2^Private Prenatal Diagnostic Centre “Diagnostica ecografica Aniello Di Meglio s.r.l.”, 80100 Naples, Italy; ^3^Department of Medical Genetics, Bambino Gesù Children's Hospital, IRCCS, 00146 Rome, Italy

## Abstract

*Background*. Prenatal diagnosis of Optiz G/BBB syndrome (OS) is challenging because the characteristic clinical features, such as facial and genitourinary anomalies, may be subtle at sonography and rather unspecific. Furthermore, molecular testing of the disease gene is not routinely performed, unless a specific diagnosis is suggested. *Method*. Both familial and ultrasound data were used to achieve the diagnosis of X-linked OS (XLOS), which was confirmed by molecular testing of *MID1* gene (Xp22.3) at birth. *Results*. Sequencing of *MID1* gene disclosed the nucleotide change c.1285 +1 G>T, previously associated with XLOS. *Conclusions*. This case illustrates current challenges of the prenatal diagnostic work-up of XLOS and exemplifies how clinical investigation, including family history, and accurate US foetal investigations can lead to the correct diagnosis.

## 1. Introduction

The development of molecular cytogenetic techniques, including array-based Comparative Genomic Hybridization (aCGH), has offered a new platform in prenatal diagnosis, in particular for evaluating fetuses with structural abnormalities [1]. However, the prenatal diagnostic work-up should always complement innovative cytogenetic and molecular techniques with the traditional investigative tools, including family history and accurate ultrasound (US) evaluation. This point is illustrated by the present case report of a prenatally diagnosed case of maternally inherited Optiz G/BBB syndrome.

## 2. Case Report

**Figure 1:**
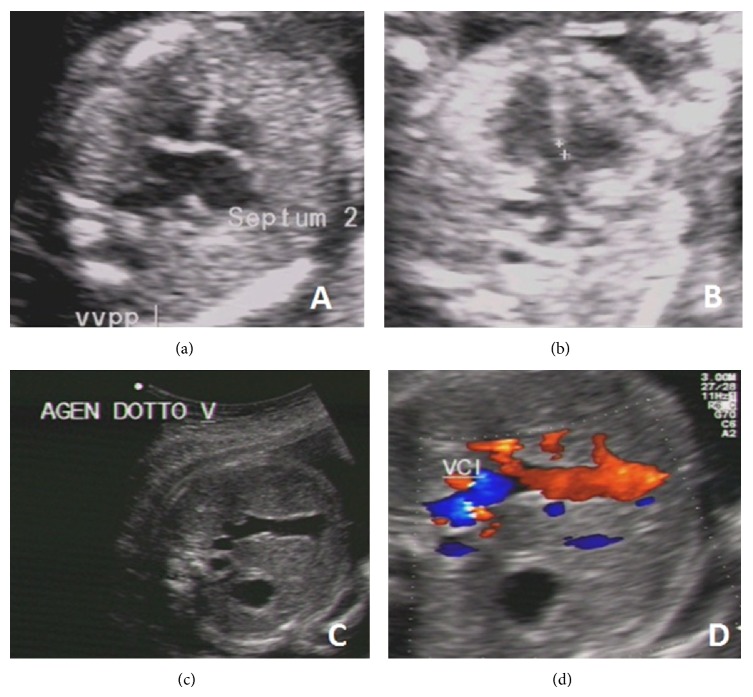
Prenatal ultrasonographic foetal heart anomalies detected at 19 ((a)* ostium primum* ASD; (b) perimembranous VSD) and 28 ((c)-(d) agenesis of the venous duct) weeks of pregnancy.

**Figure 2:**
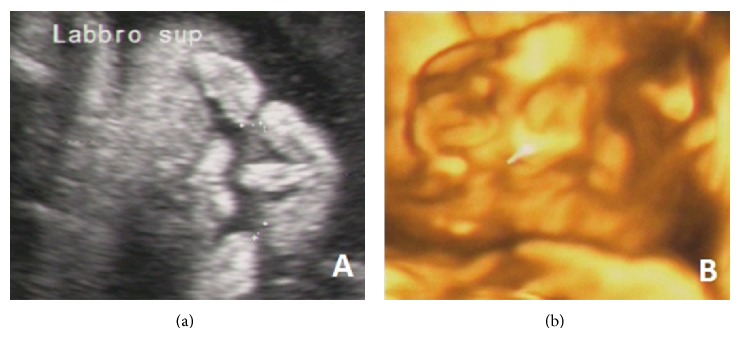
Bilateral cleft lip at 2D (a) and 3D (b) prenatal ultrasonographic scan at 19 and 28 weeks of pregnancy, respectively.

A 28-year-old nulliparous woman was referred at 19-week gestation because of suspected cardiac defect. First-trimester screening was unremarkable and nuchal translucency was 1.4 mm. Foetal ultrasound showed a male foetus with a complex congenital heart disease (CHD), including perimembranous ventricular septal defect (VSD),* ostium primum* atrial septal defect (ASD) or partial atrioventricular (AV) canal defect (Figures 1(a) and 1(b)), and Persistent Left Superior Vena Cava (PLSVC). CHD was associated with bilateral cleft lip (Figure 2(a)), a slight hypertelorism, and bilateral pyelectasis (Video) (in Supplementary Material available online at http://dx.doi.org/10.1155/2015/830108).

Amniocentesis was performed at 20 weeks and both karyotype and oligonucleotide array-based Comparative Genomic Hybridization (aCGH) (44K Chip Agilent Technologies, Waldbronn, Germany) were reported as normal.

The parents were referred to genetic counselling, which disclosed CHD (mild ASD and VSD spontaneously resolved) associated with hypertelorism and hypospadias in a 1-year-old maternal nephew. Based on family history, as well as foetal US, a segregating X-linked disorder affecting the midline structures was suspected. Following multidisciplinary counselling, during which available options and risks were discussed, the parents decided on pregnancy continuation. US follow-up at 28-week gestation confirmed bilateral cleft lip (Figure 2(b)), the slight hypertelorism, and bilateral pyelectasis. Furthermore, increased cortical echogenicity of the right kidney with upper pole caliectasis, as well as hypospadias, was detected. Foetal echocardiography confirmed the complex CHD. In addition, agenesis of venous duct, resulting in an abnormal drainage of the umbilical vein into the foetal venous circulation (Figures 1(c) and 1(d)), and a worsened heart function with cardiomegaly were found. Thus, the parents were thoroughly counseled about the potential risks related to the discovery of the additional malformations and the impaired heart function, including an early-preterm birth or an intrauterine fetal death, as well as the need to schedule delivery in a facility with neonatal and cardiac surgical intensive-care unit. Antenatal corticosteroids for accelerating fetal lung maturation were performed as well.

Preterm labour spontaneously occurred at 30 weeks. Uncontrollable cyclic premature uterine contractions and a dystocic labour required caesarean section.

**Figure 3:**
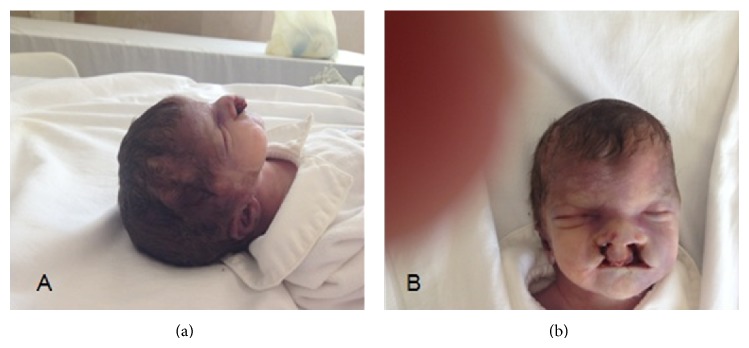
Phenotypical features of the affected newborn included a broad nasal bridge (a) and hypertelorism bilateral cleft lip and palate (b).

A malformed male neonate, weighing 1254 g (34th centile), was delivered with Apgar score of 5 at 1 minute and 6 at 5 minutes. The main abnormalities occurred along the body midline, including hypertelorism, cleft lip and palate, and hypospadia (Figure 3). Crossed fused renal ectopia, severe CHD, and imperforate anus were also present. Clinical evaluation, together with family history, corroborated the diagnosis of XLOS. Molecular testing of* MID1* gene (Xp22.2) in the proband disclosed a nucleotide change c.1285 +1 G>T, which is known to result in an abnormal exon 5 splicing [2].

The baby died 24 hours after birth of heart failure. The parents denied consent to autopsy.

**Figure 4:**
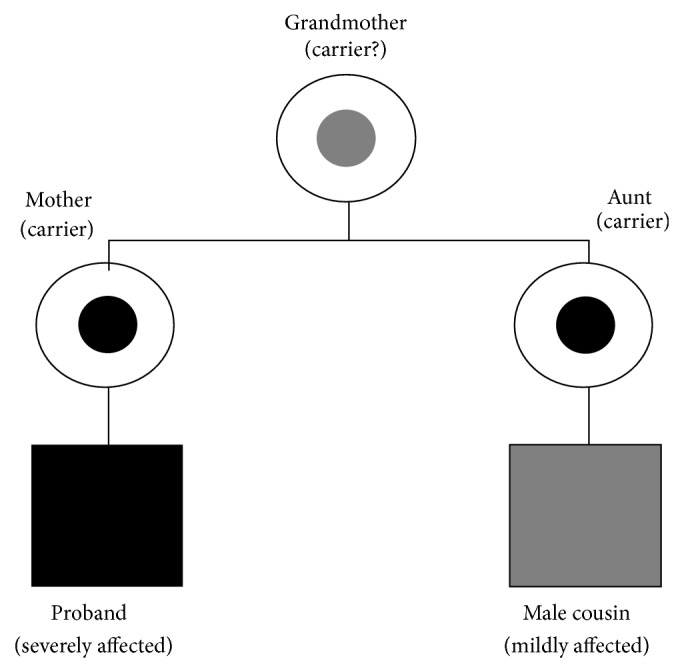
Pedigree of family in present study. Squares and circles represent males and females, respectively. Blackened symbols indicate individuals with X-linked Opitz syndrome confirmed by* MID1* mutation analysis. Gray-coloured symbols indicate individuals with a suspected mild form of X-linked Opitz syndrome, not confirmed by* MID1* mutation analysis. Small black dots within the circle indicate carrier state confirmed by* MID1* mutation analysis. Small gray dots within the circle indicate carrier state not confirmed by* MID1* mutation analysis.

Subsequent molecular testing in the mother and her sister showed that they were heterozygous for the same mutation, in the absence of any obvious XLOS features. Genetic testing was not available for the maternal nephew nor for the maternal grandmother (Figure 4).

## 3. Discussion

Opitz G/BBB syndrome (OS; MIM number 145410 and MIM number 300000), a congenital midline malformation syndrome, was first recognized in 1969 as two distinct disorders, G syndrome and BBB syndrome [3, 4]. Later on these two syndromes turned out to be a unique disease. Clinical manifestations of OS include midline defects with broad nasal bridge, hypertelorism, prominent forehead, hoarseness, low-set-posteriorly rotated ears, labiopalatine and laryngotracheal abnormalities, dysphagia and gastroesophageal reflux, central nervous system (CNS) abnormalities, causing major motor skill defects, delayed development and intellectual disability, genital anomalies, including hypospadias, cryptorchidism, and hypoplastic/bifid scrotum [5–8]. Other malformations in less than 50% of cases include CHD, in particular atrial and ventricular septal defects, patent ductus arteriosus, coarctation of the aorta, imperforate or ectopic anus, Dandy-Walker malformation, agenesis or hypoplasia of the corpus callosum, and/or cerebellar vermis [9].

OS is genetically heterogeneous with an X-linked form, (XLOS; Opitz G/BBB syndrome, type I) (OMIM 300552), and an autosomal dominant form (ADOS; Opitz G/BBB syndrome, type II) (MIM 145410) [10]. The disease gene of XLOS,* MID1*, maps to chromosome Xp22.3 [11]. Approximately 40 mutations have been reported along the entire length of the* MID1* gene in both sporadic and familial cases [5, 6, 8, 10–15].

XLOS affects 1 in 50,000 to 100,000 males. Heterozygous females can manifest mild features of OS, in particular hypertelorism, while clinical presentation in affected males is quite variable with some obligatory signs as hypertelorism, laryngotracheal abnormalities, and hypospadias [5].

The first US prenatally diagnosed XLOS was reported in 1989. The maternal family history included male deaths associated with midline abnormalities. Fetal morphology scan at 20 weeks disclosed a male foetus presenting with hypertelorism, hypospadias, and enlarged cisterna magna. The diagnosis was confirmed by pathologic examination after pregnancy termination [16]. Two additional male prenatal cases complicated by polyhydramnios, hydrops, and multiple midline malformations were diagnosed as affected by XLOS after delivery [17, 18] (Table 1).

The first prenatal genetic diagnosis of XLOS concerned a Korean who had previously delivered a son diagnosed with XLOS at 4 years of age. He presented with hypertelorism, a broad nasal bridge, a laryngeal cleft, and hypospadias. Her two subsequent pregnancies were monitored by chorionic villous sampling and amniocentesis, respectively, and disclosed in both cases a c.1798insC mutation in exon 13 of* midline protein1* (*MID1*) gene [19]. An additional prenatal case of XLOS was diagnosed by aCGH in a foetus with CHD, which turned out to be hemizygous for a 48 Kb deletion of Xp22.2, spanning the 3′UTR region of the (*MID1*) gene [1] (Table 1).

The present case illustrates the current challenges of prenatal diagnostic work-up of XLOS and raises a number of issues.

First, carrier mothers either display an unremarkable phenotype or, at most, manifest isolated hypertelorism, hampering the possibility to identify, on a clinical basis alone, females at risk, unless in the presence of one or more affected males. In our family, both the mother and the maternal sister were clinically normal and family history was negative for male deaths associated with hypertelorism, hypospadias, and cleft lip/palate [19]. An additional challenging aspect in our family was that the male maternal nephew displayed only mild features of XLOS. Accordingly, at routine anamnestic interview these characteristics could be underestimated, as in the present case, in which only after a long-lasting detailed interview the mother admitted that her male nephew had some minor congenital anomalies. This latter finding (male maternal nephew affected), together with the fact that midline defects can be transmitted in an X-linked manner, allowed us the suspicion of a midline-X-linked disorder.

Second, the distinguishing features of XLOS, as hypertelorism and hypospadias, are rather subtle and quite often are not detected by prenatal US. The wide spectrum of possible associated defects makes the clinical diagnosis quite difficult. In our case, the main clinical features, found at second trimester US, were bilateral cleft lip and CHD, which occur in less than 50% of XLOS patients [8]. However, accurate US scans disclosed also some subtle diagnostic abnormalities, that is, a slight hypertelorism, which were used as handles to suggest the correct diagnosis.

In conclusion, we experienced a difficult prenatal diagnostic work-up of XLOS, which was successfully accomplished based on integration of family history, US findings, and molecular test.

As a proper prenatal diagnosis of causative foetal disease is desirable for suitable prenatal as well as neonatal care, it is worth clinically accumulating evidence considering the integration of data coming from anamnesis and ultrasonographic findings, in order to obtain final diagnosis by specific molecular analysis. Such findings enable proper genetic counselling and allow parents to make appropriate decisions in their pregnancy. It also carries important implications to the family and future pregnancies.

## Supplementary Material

The clip show the case of a 19-week male foetus with a complex congenital heart disease (CHD), including perimembranous ventricular septal defect, ostium primum atrial septal defect and Persistent Left Superior Vena Cava. CHD was associated with bilateral cleft lip, a slight hypertelorism, and bilateral pyelectasis. These midline defects, together with the family hystory were suspicious for Opitz syndrome.

## Figures and Tables

**Table 1:** List of reported cases of prenatally detected maternally inherited Opitz syndrome (modified from Cheng et al., 2014 [1]).

	Prenatal ultrasonographic features	Type of prenatal diagnosis	Delivery gestational age and birth weight	Additional postnatal phenotypical findings
Patton et al., 1986 [17]	(i) Hydrops (ii) Polyhydramnios	Ultrasonography	37 weeks 3012 g	(i) Posteriorly rotated ears (ii) Loose skin folds in neck (iii) Inguinal hernia (iv) Anteriorly placed anus

Hogdall et al., 1989 [16]	(i) Hypertelorism (ii) Enlarged cisterna magna	Ultrasonography	TOP II trimester	(i) Posteriorly rotated low-set ears (ii) Imperforate anus

Cho et al., 2006 [19]	NA	Cytogenetic analysis	TOP I trimester	NA

Tajima et al., 2010 [18]	(i) Polyhydramnios (ii) Cleft lip	Ultrasonography	35 weeks 2076 g	(i) Hypertelorism (ii) Cleft palate (iii) Hypospadias (iv) Dysphagia

Cheng et al., 2014 [1]	(i) Congenital heart disease	Ultrasonography + cytogenetic analysis	TOP II trimester	(i) Absence of the corpus callosum

TOP: termination of pregnancy.

NA: not available.
